# Real-world performance of myositis antibody panels: a single-centre experience

**DOI:** 10.1093/rheumatology/keag343

**Published:** 2026-06-26

**Authors:** Marina Dueñas-Ochoa, Roni Meidan, João Francisco Abrantes, Emily Apsley, David Isenberg

**Affiliations:** Department of Rheumatology, La Princesa University Hospital, Madrid, Spain; Department of Rheumatology, Tel Aviv Sourasky Medical Center, Gray Faculty of Medical and Health Sciences, Tel Aviv University, Tel Aviv, Israel; Department of Internal Medicine, Santa María University Hospital, Lisbon, Portugal; TDL Pathology, London, UK; Centre for Ageing, Rheumatology and Regenerative Medicine, Division of Medicine, University College London, London, UK

Rheumatology key message

- Myositis antibodies have low positive predictive value but high negative predictive value in routine practice.

Dear Editor, The increasing availability of panels of myositis autoantibodies is an exciting development in the evaluation of inflammatory muscle diseases. In order to assess how frequently these antibodies assist the physician in coming to a diagnosis in a real-world setting, we conducted a retrospective review of all requests for a comprehensive myositis autoantibody panel at our major teaching hospital over the course of 2025.

A total of 1047 requests were identified in an ethnically diverse population: Caucasian (36.3%), Black (9.2%), South Asian (9.76%), Hispanic (0.2%) and unspecified (44.4%). Myositis-specific antibodies (MSAs) and myositis-associated antibodies (MAAs) were tested using the EUROLINE Autoimmune Inflammatory Myopathies 16 Ag (IgG) line blot immunoassay (LIA), which includes anti-Jo-1, anti-OJ, anti-EJ, anti-PL-7, anti-PL-12, anti-SRP, anti-SAE, anti-NXP2, anti-MDA5, anti-TIF1γ, anti-Mi2α, anti-Mi2β, anti-PM-Scl75, anti-PM-Scl100, anti-Ku and anti-Ro52. Anti-HMGCR antibodies were not included in the panel. Results were reported by signal intensity as negative (<11), weakly positive (11–25) and positive (≥26). Weak positives were considered negative in our study. In patients with repeated testing, only the first sample was analysed. Electronic medical records were reviewed to collect demographic and clinical data, including test indication, requesting specialty and final diagnosis as determined by the treating physician. In antibody-positive cases, additional data on laboratory findings and complementary tests (electromyography, muscle MRI and biopsy) were obtained. Diagnoses were recorded irrespective of their timing relative to antibody testing.

Patients were categorised into three groups: idiopathic inflammatory myopathy (IIM), other autoimmune rheumatic diseases (ARDs) and non-IIM/ARD conditions. IIM cases were further subclassified into dermatomyositis (DM), anti-synthetase syndrome (ASyS), polymyositis (PM), overlap myositis (OM), immune-mediated necrotising myopathy (IMNM), inclusion body myositis (IBM) and unclassified IIM where insufficient data were available. Where available, classification criteria were retrospectively applied and IIM diagnosis was confirmed according to the 2017 EULAR/ACR criteria [1] for DM, PM, OM and ASyS; ENMC criteria for IMNM and IBM [2, 3]; and Connors *et al.* [4] proposed criteria for ASyS. The ARD group included SLE, Sjögren’s disease, systemic vasculitis, rheumatoid arthritis, systemic sclerosis and undifferentiated autoimmune rheumatic disease. The remaining group comprised patients with alternative diagnoses or in whom IIM was reasonably excluded. Cases with insufficient clinical data were excluded. We also recorded the presence of interstitial lung disease (ILD), defined by an expert pulmonologist, and IIM-associated cancer, defined as malignancy occurring within 3 years before or after IIM diagnosis [5], where sufficient follow-up time was available to assess this period. Measures of central tendency (mean or median) and dispersion (s.d. or interquartile range) were used to describe quantitative variables, and frequencies and percentages for qualitative variables.

The final cohort comprised 932 patients (52.8% female), of whom 242 patients (25.9%) tested positive for at least one MSA/MAA. Patient exclusion details are provided in Supplementary Fig. S1. Only 68 (7.3%) patients in the overall group fulfilled criteria for probable or definite IIM. In all these patients, the diagnosis of IIM was confirmed by an expert rheumatologist/neurologist. Among MSAs, anti-Mi2β was the most frequently detected (14.9%), followed by anti-PL-7 (12.4%) and anti-Jo-1 (5.8%). Among the MAAs, anti-Ro52 (31.8%) and anti-PM-Scl75 (14.9%) were predominant. Isolated MSA positivity occurred in 42.6% cases, while 5.8% presented multiple MSAs and 9.9% showed MSA/MAA overlap. Requests for myositis antibody testing were mainly initiated by respiratory medicine (52.9%), followed by rheumatology (18.9%) and neurology (14.9%). Suspected ILD (44.5%), objective muscle weakness (12.9%) and systemic symptoms or generalized myalgia (17.2%) were the primary clinical indications.

Among patients with positive MSA/MAA, only 36 (14.9%) were diagnosed with IIM, whereas 23.1% met criteria for other ARDs. ILD (either isolated or associated with systemic disease) was present in 45.5% of positive cases and 29.3% had alternative diagnoses. Further clinical data are summarized in Table 1.

**Table 1:** Diagnoses and clinical characteristics of the patients.

All patients tested for myositis antibodies	Positive patients (*n* = 242)	Negative patients (*n* = 690)	Total (*n* = 932)
Final diagnosis, *n* (%)			
IIM	36 (14.9)	32 (4.6)	68 (7.3)
Definitive	26 (10.7)	29 (3.8)	55 (5.9)
Probable	10 (4.1)	3 (0.4)	13 (1.4)
Other ARD	56 (23.1)	52 (7.5)	108 (11.6)
ILD/IPAF	110 (45.5)	210 (30.4)	320 (34.4)
Alternative diagnoses, *n* (%)	71 (29.3)	411 (59.6)	482 (51.7)

Patients with IIM	Positive with IIM (*n* = 36)	Negative with IIM (*n* = 32)	Total with IIM (*n* = 68)
Female, *n* (%)	26 (72.2)	16 (50)	42 (61.8)
Age at diagnosis, years, mean (s.d.)	41.4 (±20)	40.2 (±25.8)	40.9 (±22.7)
Time of follow-up, years, median (IQR)	1.5 (1–7)	4 (1.8–8.5)	2.5 (1–7)
Subtype of IIM, *n* (%)			
DM	10 (27.8)	11 (34.4)	21 (30.9)
ASyS	5 (13.9)	0 0	5 (7.4)
IMNM	3 (8.3)	4 (12.5)	7 (10.3)
IBM	4 (11.1)	7 (21.9)	11 (16.2)
PM	0 0	1 (3.1)	1 (1.5)
OM	7 (19.4)	5 (15.6)	12 (17.6)
Unclassified IIM	7 (19.4)	4 (12.5)	11 (16.2)
Clinical manifestations, *n* (%)			
Proximal muscle weakness	34 (94.4)	26 (81.3)	60 (88.2)
Skin manifestations	14 (38.9)	15 (46.9)	29 (42.6)
Joint involvement	12 (33.3)	10 (31.2)	22 (32.2)
Dysphagia	13 (36.1)	8 (25)	21 (30.9)
Calcinosis	2 (5.6)	1 (3.1)	3 (4.4)
Associated ILD, *n* (%)	12 (33.3)	3 (9.4)	15 (22.1)
Cancer related to IIM, *n* (%)	3 (8.3)	1 (3.1)	4 (5.9)
Antibodies, *n* (%)			
MSAs	4 (11.1)		
JO-1, n (%)	1 (2.8)		
EJ, n (%)	0 0		
OJ, n (%)	2 (5.6)		
PL12, n (%)	1 (2.8)		
PL7, n (%)	2 (5.6)		
Mi2α, n (%)	2 (5.6)		
Mi2β, n (%)	4 (11.1)		
NXP2, n (%)	4 (11.1)		
MDA5, n (%)	2 (5.6)		
SRP, n (%)	3 (8.3)		
SAE, n (%)	1 (2.8)		
TIF1g, n (%)	2 (5.6)		
MAAs			
Ro52, n (%)	20 (55.6)		
PM-Scl-75, n (%)	8 (22.2)		
PM-Scl-100, n (%)	4 (11.1)		
Ku, n (%)	1 (2.8)		
Elevated CK at diagnosis, *n* (%)			
Yes	22 (61.1)	25 (78.1)	47 (69.1)
No	6 (16.7)	0 0	6 (8.8)
Unknown	8 (22.2)	7 (21.9)	15 (22)
Electromyography, *n* (%)			
Positive	9 (25)	13 (40.6)	22 (32.4)
Negative	5 (13.9)	2 (6.3)	7 (10.3)
Not done	13 (36.1)	9 (28.1)	22 (32.4)
Unknown	9 (25)	8 (25)	17 (25)
Muscle biopsy, *n* (%)			
Positive	9 (25)	16 (50)	25 (36.8)
Negative or not diagnostic	6 (16.7)	1 (3.1)	7 (10.3)
Not done	12 (33.3)	11 (34.4)	23 (33.8)
Unknown	6 (16.7)	4 (12.5)	10 (31.3)
Muscle MRI, *n* (%)			
Suggestive	23 (63.9)	22 (68.8)	45 (66.2)
Not suggestive or unspecific	2 (5.6)	2 (6.3)	4 (5.9)
Not done	5 (13.9)	3 (9.4)	8 (11.8)
Unknown	6 (16.7)	4 (12.5)	10 (14.7)

IPAF: interstitial pneumonia with autoimmune features; CK: creatine kinase.

The positive predictive value (PPV) for having any positive myositis antibody in our cohort was 14.9% for IIM, considerably lower than previously reported figures [6, 7], reflecting a high proportion of false-positive results. In addition, the overall sensitivity and specificity of the test were limited (52.9% and 76.1%, respectively) and the positive likelihood ratio was low (LR^+^ 2.21), indicating that a positive result confers only a modest increase in the post-test probability of disease. These findings suggest that MSA/MAA positivity, particularly in unselected populations and in the absence of cardinal symptoms, should be interpreted with caution. Crucially, almost half of patients with confirmed IIM in our cohort were seronegative; consequently, the negative likelihood ratio (LR^−^) was 0.62. This further highlights that reliance on antibody negativity is insufficient to reliably rule out myositis and may result in a substantial proportion of missed diagnoses. Notably, these antibodies were also identified in a range of other ARDs (PPV 23.1%) and ILD (PPV 45.5%), underscoring their limited disease specificity for IIM.

Limitations of this study include its retrospective design and the relatively short follow-up period for some patients, potentially limiting the assessment of long-term outcomes. Key strengths include the large sample size and the inclusion of a heterogeneous population, including patients with and without suspected inflammatory myopathy. Furthermore, the assessment of individuals with negative antibody results provides insights into the clinical characterization of seronegative presentations within this cohort. In conclusion, positive results of this array of tests should be interpreted with particular care and correlation with clinical findings is essential to avoid potential misclassification or overdiagnosis.

## Supplementary Material

keag343_Supplementary_Data

## Data Availability

Data will be shared upon reasonable request.
