# Supplementary material for: Real-world performance of myositis antibody panels: a single-centre experience
Source: Rheumatology (Oxford). 2026 Jun 26;65(7):keag343. doi: 10.1093/rheumatology/keag343 (PMC13378450; doi:10.1093/rheumatology/keag343)
Supplement: keag343_Supplementary_Data [file keag343_supplementary_data.docx]

**Supplementary material:**


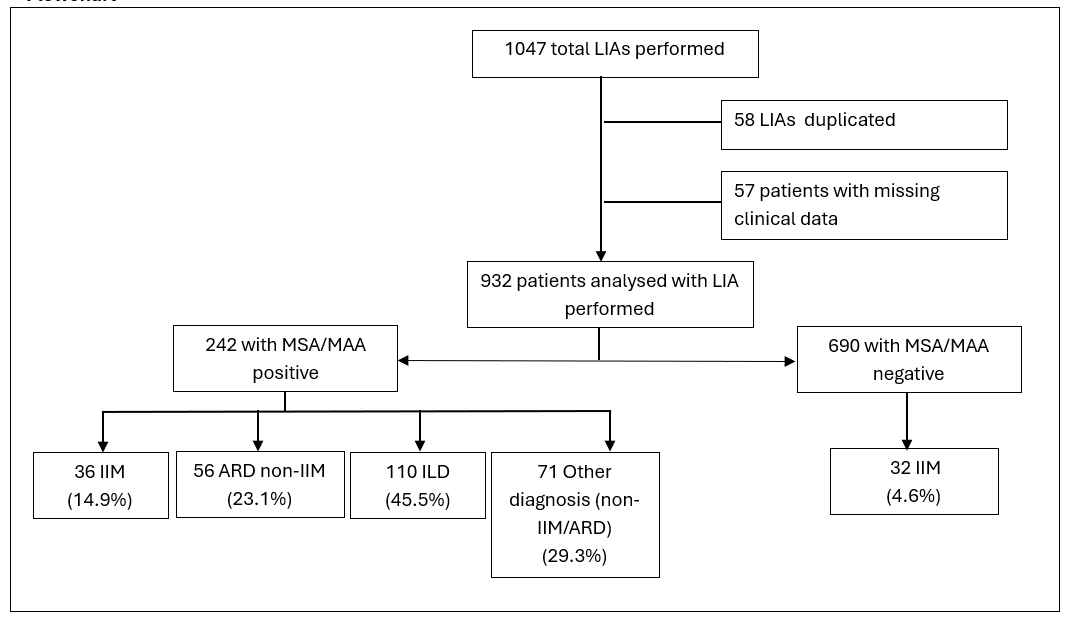


**Supplementary Figure S1. Study population and final diagnoses.**

**Abbreviations**: LIA: line immunoassay; MSAs: myositis-specific autoantibodies; MAAs: myositis-associated autoantibodies. IIM: idiopathic inflammatory myopathies; ARD: autoimmune rheumatic diseases; ILD: interstitial lung disease.
